# Residential proximity to major roadways and hearing impairment in Chinese older adults: a population-based study

**DOI:** 10.1186/s12889-023-17433-6

**Published:** 2023-12-08

**Authors:** Xingxing Chen, Jun Wang, Xian Zhang, Gui Xiao, Siran Luo, Lei Liu, Weijia Kong, Xiaomin Zhang, Lijing L. Yan, Sulin Zhang

**Affiliations:** 1 School of Public Health, Wuhan University, Wuhan, China; 2 Global Health Research Center, Duke Kunshan University, Kunshan, China; 3 National Institute for Stroke and Applied Neurosciences, Auckland University of Technology, Auckland, New Zealand; 4 Department of Otorhinolaryngology, Union Hospital, Tongji Medical College, Huazhong University of Science and Technology, Wuhan, China; 5 Institute of Otorhinolaryngology, Union Hospital, Tongji Medical College, Huazhong University of Science and Technology, Wuhan, China; 6 Xiangya School of Nursing, Central South University, Changsha, China; 7 Department of Health Management, The Third Xiangya Hospital, Central South University, Changsha, China; 8 The First People’s Hospital of Kunshan, Suzhou, China; 9 Department of Occupational and Environmental Health, State Key Laboratory of Environmental Health for Incubating, School of Public Health, Tongji Medical College, Huazhong University of Science and Technology, Wuhan, China; 10 Duke Global Health Institute, Duke University, Durham, United States of America; 11 Institute for Global Health and Management, Peking University, Beijing, China

**Keywords:** Hearing impairment, Major roadways, Traffic-related air pollution, Traffic noise exposure, Older adults

## Abstract

**Background:**

With rapid urban sprawl, growing people are living in the vicinity of major roadways. However, little is known about the relationship between residential proximity to major roadways and hearing impairment (HI).

**Methods:**

We derived data from the 2018 wave of the Chinese Longitudinal Healthy Longevity Survey, and included 13,775 participants aged 65 years or older. Multivariate logistic regressions were employed to examine the association between residential proximity to major roadways and HI. The effects of corresponding potentially modifiable factors were studied by three-way interaction analyses. Sensitivity analyses were performed to verify the robustness of the results.

**Results:**

The prevalence of HI was 38.3%. Participants living near major roadways were more likely to have a higher socioeconomic status. An exposure-response relation between residential proximity to major roadways and HI was observed (*P*_trend_ < 0.05). Compared with individuals living > 300 m away from major roadways, the adjusted odds ratios (OR) were 1.07 (95% CI: 0.96–1.24), 1.15 (95% CI: 1.07–1.34), and 1.12 (95% CI: 1.01–1.31) for those living 101–200 m, 50–100 m, and < 50 m away from the roadways, respectively. Particularly, the association was more pronounced among individuals exposed to carbon monoxide (CO) pollution or opening windows frequently (*P*_interaction_ < 0.05). Three-way interaction analyses confirmed that participants exposed to CO pollution and frequently leaving windows open had the highest OR of 1.73 (95% CI: 1.58–1.89).

**Conclusions:**

This nation-wide cohort study suggested that residential proximity to major roadways was significantly associated with an increased exposure-response risk of HI in Chinese older adults. Exposure to CO pollution and opening windows frequently might strengthen the relations.

**Supplementary Information:**

The online version contains supplementary material available at 10.1186/s12889-023-17433-6.

## Key Points

• An inverse exposure-response relation between residential proximity to major roadways and hearing impairment was observed.

• Exposure to carbon monoxide pollution and opening windows frequently might synergistically intensify this relation.

• Our findings supported city planners to consider locating housing developments away from the most heavily trafficked major roadways on a public policy level.

**Graphical Abstract:**

**Supplementary Information:**

The online version contains supplementary material available at 10.1186/s12889-023-17433-6.

## Graphical abstract

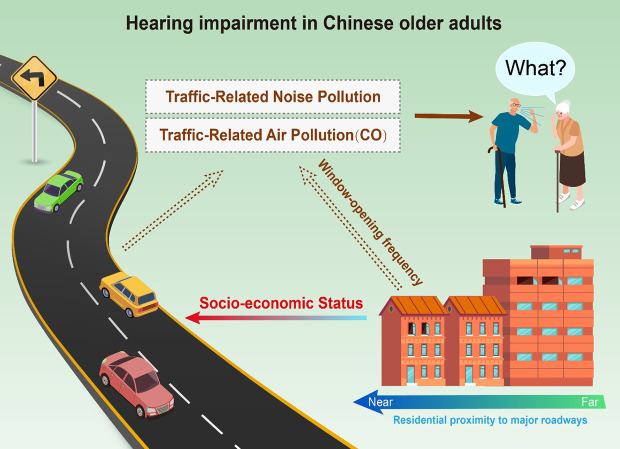

## **Introduction**

Hearing impairment (HI) afflicts approximately one-third of adults over 65 years old [1] and its prevalence roughly doubles with each passing decade, currently being the third leading chronic condition among older adults [2, 3]. Unfortunately, most people with HI go undiagnosed or untreated for many years, resulting in a series of unfavorable outcomes, including disability [4], dementia [5], and mortality [6], among others. Thus, it is an urgent task to identify the risk factors of HI and take measures to lower its burden on individuals and the society at large.

Despite global endeavor, progresses in the research on the risk factors of HI remain limited. Moreover, concern is mounting that exposures to road noise [7] and traffic-related air pollution [8] might be high risks of HI. Existing evidence suggests that long-term exposure to traffic noise poses a higher risk for HI than sudden high-level noise [9]. Apart from direct effects on the hearing function, continuous road traffic noise also brings about psychological and physiological stresses, leading to an increased susceptibility to noise trauma [10]. Similarly, traffic emissions represent a complex mixture of air pollutants, such as particulate matters (PM2.5 and PM10) [11], carbon monoxide (CO) [12], and so on. Each of these components, along with their secondary by-products can adversely affect hearing function [13].

Residential proximity to major roads represents a heightened exposure to road noise and automobile exhaust [14]. Studies have proven that living close to major roadways might contribute to a wide array of adverse outcomes [15, 16], and systemic inflammatory and oxidative stress responses might underlie such causal relation [17]. The ear is an organ highly subject to oxidative stresses, which result in damage of hair cells in the Corti’s organ within the cochlea [18]. Given this susceptibility, residing near a major roadway may also exert a negative impact on hearing function. To date, however, no studies have specifically evaluated whether living in proximity to major roadways is associated with HI. Moreover, the adverse impact of HI is more pronounced among older adults, posing disproportionately higher burden on both individuals and the healthcare system [19]. It is imperative, therefore, to identify risk factors for this disease to inform policy-making and healthcare practices.

Against this backdrop, we aimed to use cross-sectional data from the most recent 2018 Chinese Longitudinal Healthy Longevity Survey (CLHLS) wave, to estimate: (i) whether residential proximity to major roadways (a proxy of exposure to traffic) was associated with HI in an exposure-response manner among Chinese older adults; (ii) whether the association was modified by other factors. To our knowledge, this is the first study to examine the issue in low and middle-income countries (LMICs) and it utilized one of the largest datasets in the world.

## Materials and methods

### Study population

We used data from the CLHLS, an ongoing program which has been implemented since 1998, with follow-ups conducted every 2–3 years. The CLHLS was implemented in randomly selected counties and cities from half of all counties and cities in 23 of the 31 provinces of China, covering over 85% of China’s population. As all adults aged 65 to 99 were randomly selected, the CLHLS sample can well represent older adults in China. Details of this survey have been published elsewhere [20]. This study was reported according to the STROBE guidance for reporting observational studies (Checklist in Table A1) [21].

**Fig. 1:**
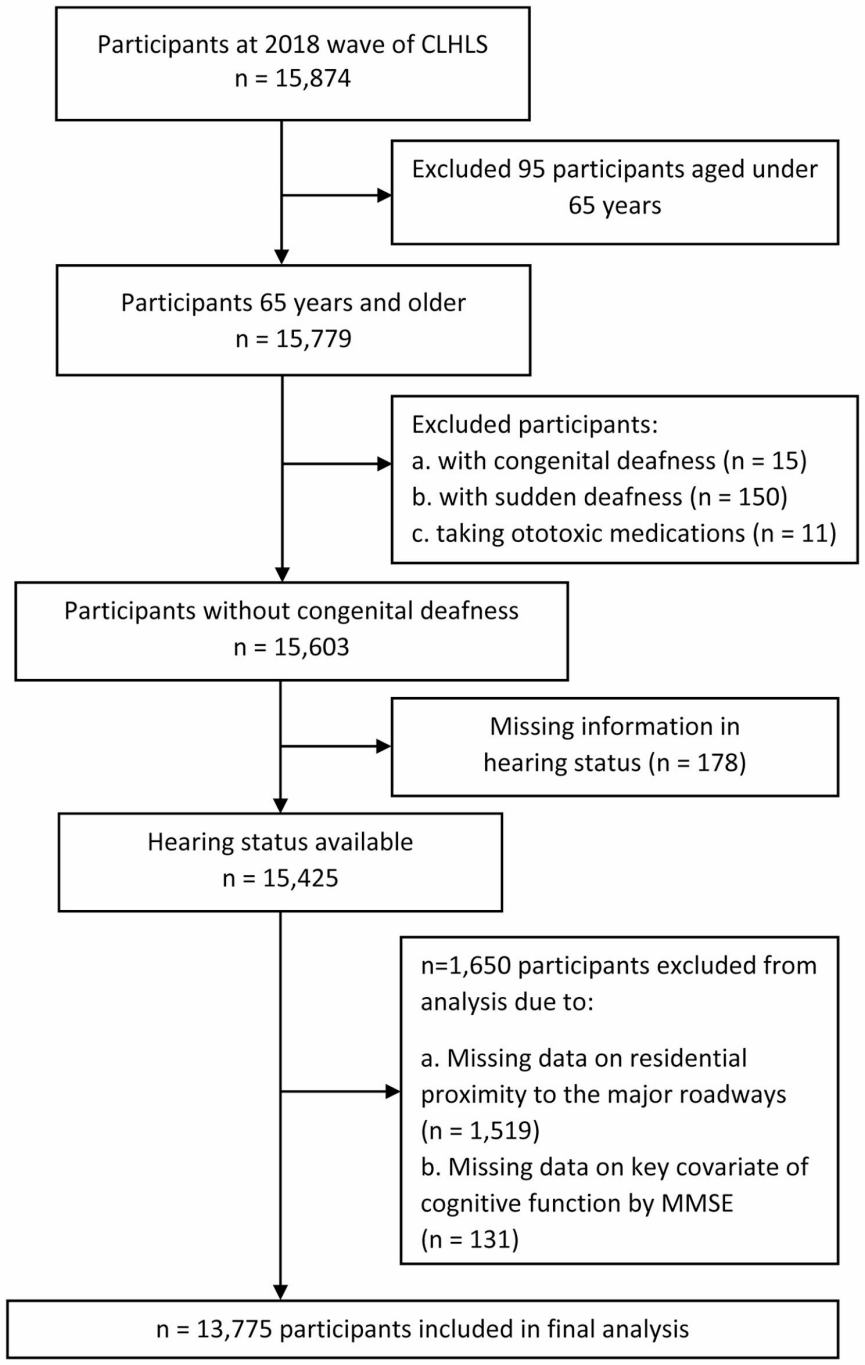
The flow chart of study population

In the present study, we used data of the 2018 wave of CLHLS, involving 15,874 participants. First, we excluded 95 participants aged under 65 years. Then, participants with congenital deafness (n = 15) and sudden deafness (n = 150) were removed from our analysis. Additionally, considering the interference of ototoxic drugs on the hearing status, participants who were taking ototoxic medications were eliminated from the main analysis (n = 11). Next, data on hearing status and residential proximity to major roadways were collected. After exclusion of 178 participants without data on hearing status, 1519 without residential information, and 131 without key covariables (cognitive function, as assessed by Chinese version of the Mini-Mental State Examination [MMSE]), 13,775 participants aged 65 years or over remained. The flow chart of study population is shown in Fig. 1.

### Residential proximity to major roadways

Residential proximity to major roadways was collected by asking subjects to respond to the question “How many meters is your home horizontally from the main/major traffic artery/line?” The major traffic artery/line was defined as “dual carriageway with at least 4 lanes”. This question had five choices as answers: ‘<50’, ‘50–100’, ‘101–200’, ‘201–300’, and ‘>300’ (in meters) .

### Assessment of hearing impairment (HI)

Data on HI were obtained by asking the participants to answer the question: “Do you have any difficulty with your hearing?”, with two possible answers available: “Yes” and “No”. “Yes” was coded as having HI, while “No” as not having HI [22]. A systematic review revealed that self-reported hearing status could be used for an epidemiological study if audiometric measurement was feasible [23]. The reliability of self-reported hearing status in CLHLS has been confirmed in previous studies [24, 25].

### Covariates

On the basis of previous studies [26–28], we categorized potential confounders into three sets: demographic features, health-related behaviors and metrics, diseases and air pollution-related data. Demographic features included age, sex, marital status (currently married and living with spouse, separated/divorced/never married, or widowed), years of education (0, 1–6, and > 6 years), and residence (rural vs. urban). Health-related behaviors and metrics included smoking status (current smoking vs. non-current or non-smoking), drinking status (current drinking vs. non-current or non-drinking), physical activity (never, former, or current), dietary diversity (low, moderate, or high), cognitive impairment (without vs. with) and body mass index (BMI). To further mitigate the potential interference of leisure noise exposure with the robustness of our results, we additionally made adjustment for the frequency of eight kinds of leisure activities in our main analysis. We utilized the PM2.5 and CO concentration averages during the 12 months preceding the surveyed months as the exposure values. Air pollution-related data involved particulate matters (annual average continuous PM2.5), annual average concentration of CO and NO_2_ (continuous), household ventilation (everyday vs. occasionally, rarely or never), and monthly average temperature (continuous). Detailed descriptions of annual estimates of air pollutants have been provided in ***Supplementary file***. We assumed that all participants living in this city/area were under the same exposure conditions in all analyses. The five self-reported chronic diseases included: diabetes, hypertension, heart diseases, stroke, and cancer (with vs. without). All self-reported information was harvested through a face-to-face home interview by trained team members. Descriptions and definitions of covariates are detailed in the supplementary file (Appendix methods).

### Statistical analysis

Multiple imputation (MI) was used to impute missing data for covariates given that the missing data accounted for less than 10% and were missing/lost at random [29]. We employed predictors of some demographics, such as sex, age, place of residence to impute the missing values of key covariates. We created 10 imputed datasets and performed pooled statistical inference [30].

Baseline characteristics of the participants were presented as counts, with percentages for categorical variables and mean ± standard deviation (SD) for continuous variables. ANOVA for continuous variables, chi-square tests for binary variables, and Kruskal-Wallis *H* test for categorical variables were applied to compare the differences between distance categories. We established four multivariable logistic regression models to evaluate the association between residential proximity to major roadways and HI: Model 1: age-sex model, Model 2: further adjustment for residency, education level, marital status, smoking status, drinking status, physical activity, leisure activity, dietary diversity, cognitive function, and BMI based on Model 2, Model 3: further adjustment for household ventilation, annual average CO concentration (continuous), annual average PM2.5 concentration (continuous), annual average NO_2_ concentration (continuous), monthly average temperature based on Model 2, Model 4 (fully-adjusted model): further adjustment for the aforementioned five diseases (diabetes, hypertension, heart disease, stroke and cancer) based on Model 3.

In order to assess disparities across different populations and modifying effects, residential proximity to major roadways was taken as a dichotomous variable (> 100 m or ≤ 100 m) in subgroup and interaction analysis according to a prior study [26]. In subgroup analyses, we examined whether the association between the residential proximity to major roadways and HI differed by age, sex, residency, education years, annual family income, cognitive function, physical activity, frequency of window opening, annual average CO concentration, monthly average temperature. Moreover, CO is considered to be ototoxic and the risk of HI was higher among participants exposed to CO [12]. Thus, we additionally tested the modifying effects of CO concentration and frequency of window opening, and three-way interaction analysis was performed to look into the association between residential proximity to major roads, window-opening, CO concentration, and HI by using multiplicative interaction methods. Before the three-way interaction analysis, we also performed an additional *t*-test to assess the correlation between window-opening and CO concentration, ensuring the independence of each factor in the interaction.

We conducted several sensitivity analyses to verify the robustness of the results of Model 4. First, we excluded participants who changed their residence within five years to determine whether short-term exposure would affect the observed associations [31]. Second, we removed participants with severe cognitive impairment (MMSE score lower than 18), to rule out the recall bias when reporting the distance to major roadways and the potential bias when evaluating hearing function [32]. With regard to the covariates used in our main models, we utilized robust Poisson regression to repeat our main analyses [33]. We also eliminated participants with the five diseases (diabetes, hypertension, heart disease, stroke and cancer) to see whether the significance level of the association would change. Finally, we conducted the primary analysis repeatedly, adjusting for age as a binomial term to estimate the age-related effects on HI.

The MI was conducted using R package ‘mice’ [34]. A two-tailed P-value of less than 0.05 was considered statistically significant. All analyses were performed using R software, version 4.1.1 (The R Foundation for Statistical Computing, Vienna, Austria).

## Results

### Characteristics of study participants

Table 1 shows the characteristics of participants in terms of the distance from residence to major roadways. The median age of participants was 85.0 years (range: 65–117 years) and more than 50% of them (55.9%) were women. The prevalence of self-reported HI stood at 38.3%. A total of 2494 participants (18.1%) lived within 50 m from a major roadway; 2128 (15.5%) resided 50 to 100 m, 1438 (10.4%) 101 to 200 m, 1305 (9.5%) 201 to 300 m, and 6410 (46.5%) > 300 m away from major roadways. Participants dwelling closer to major roadways were more likely to be younger, live in urban areas, have a higher level of education, receive higher annual family incomes, exercise frequently, be free of cognitive impairment, and expose to CO pollution (all *P* < 0.05). Participants living closer to major roadways opened the windows frequently (*P* < 0.001). More information on participants’ characteristics is listed in Table A2.

**Table 1:** Characteristics of the study participants by the different groups of horizontal distance to the major roadways

	Distance From Residence to Major Roadways (m)						*P*_trend_ Value
	**< 50** **(n = 2494)**	**50–100** **(n = 2128)**	**101–200** **(n = 1438)**	**201–300** **(n = 1305)**	**> 300** **(n = 6410)**	**Total** **(n = 13,775)**	
**Age**, years, mean (SD)	84.8 (11.5)	85.2 (11.6)	85.3 (11.6)	85.6 (11.7)	85.7 (11.6)	85.2 (11.6)	0.001
**Hearing function**, N (%)							0.005
Impaired	1007 (40.4)	827 (38.9)	524 (36.4)	515 (39.5)	2396 (37.4)	5269 (38.3)	
Non-impaired	1487 (59.6)	1301 (61.1)	914 (63.6)	790 (60.5)	4014 (62.6)	8506 (61.7)	
**Sex**, N (%)							0.347
Men	1103 (44.2)	955 (44.9)	651 (45.3)	564 (43.2)	2800 (43.7)	6073 (44.1)	
Women	1391 (55.8)	1173 (55.1)	787 (54.7)	741 (56.8)	3610 (56.3)	7702 (55.9)	
**Residency**, N (%)							< 0.001
Rural	1013 (40.6)	672 (31.6)	462 (32.1)	482 (36.9)	3483 (54.3)	6112 (44.4)	
Urban	1481 (59.4)	1456 (68.4)	976 (67.9)	823 (63.1)	2927 (45.7)	7663 (55.6)	
**Education attainment**^**a**^, N (%)							
Illiterate	1101 (44.1)	960 (45.1)	724 (50.3)	620 (47.5)	3335 (52.1)	6740 (48.9)	< 0.001
Primary school	558 (22.4)	414 (19.5)	302 (21.0)	257 (19.7)	1573 (24.5)	3104 (22.5)	< 0.001
Middle school or higher	835 (33.5)	754 (35.4)	412 (28.7)	428 (32.8)	1502 (23.4)	3931 (28.6)	< 0.001
**Family annual income**, N (%)							
< 30,000	1053 (42.2)	795 (37.4)	566 (39.4)	553 (42.4)	3960 (61.8)	6927 (50.3)	0.021
30,000–50,000	827 (33.2)	772 (36.3)	531 (36.9)	517 (39.6)	1666 (26.0)	4313 (31.3)	0.09
> 50,000	614 (24.6)	561 (26.3)	341 (23.7)	235 (18.0)	784 (12.2)	2535 (18.4)	< 0.001
**Physical activity**, N (%)							
Never	1420 (56.9)	1200 (56.4)	796 (55.3)	730 (55.9)	4249 (66.3)	8395 (60.9)	< 0.001
Former	182 (7.3)	196 (9.2)	152 (10.6)	144 (11.0)	513 (8.0)	1187 (8.6)	
Current	892 (35.8)	732 (34.4)	490 (34.1)	431 (33.1)	1648 (25.7)	4193 (30.5)	
**Leisure activity**^**c**^, N (%)							0.26
Low	2238 (89.7)	1899 (89.2)	1280 (89.0)	1159 (88.8)	5791 (90.3)	12,367 (89.8)	
High	256 (10.3)	229 (10.8)	158 (11.0)	146 (11.2)	619 (9.7)	1408 (10.2)	
**Cognitive impairment**^**d**^, N (%)							0.006
Without	1489 (59.7)	1213 (57.0)	860 (59.8)	761 (58.3)	3639 (56.8)	7962 (57.8)	
With	1005 (40.3)	915 (43.0)	578 (40.2)	544 (41.7)	2771 (43.2)	5813 (42.2)	
**Windows opening**^**e**^, N (%)							< 0.001
Everyday or occasionally	2265 (90.8)	1852 (87.0)	1224 (85.1)	1181 (90.5)	5490 (85.6)	12,012 (87.2)	
Rarely or never	229 (9.2)	276 (13.0)	214 (14.9)	124 (9.5)	920 (14.4)	1763 (12.8)	
**CO** (ppm), mean (SD)	0.86 (0.15)	0.85 (0.14)	0.84 (0.14)	0.83 (0.14)	0.83 (0.15)	0.85 (0.14)	< 0.001
**Temperature** (℃), mean (SD)	19.22 (8.33)	20.52 (8.02)	20.17 (8.51)	20.28 (8.37)	20.33 (8.31)	19.81 (8.34)	< 0.001

^a^Defined by years of schooling. Illiterate: school years = 0; Primary school: school years = 1–5; Middle school or higher: school years > 5

^b^Dietary diversity was categorized as: Low (Dietary diversity score < 4); median (Dietary diversity score between 4 to 6); high (Dietary diversity score > 6)

^c^A leisure activities score was calculated by eight kinds of activities (housework, fieldwork, gardenwork, reading, pets, Mahjong, tv, social-activity) and we scored each activity 1 for ‘never’, 2 for ‘sometimes’, and 3 for ‘almost every day’. The score ranged from 6 to 24 with higher scores indicate more frequent leisure activities. Low leisure activity level was defined by the score less than 14

^d^Cognitive function was assessed by Chinese version of the Mini-Mental State Examination (MMSE) that included 24 items, covering seven subscales. The MMSE score ranges from 0 to 30 and higher scores represent better cognitive function. Cognitive impairment was defined as MMSE scores lower or equal to 25

^e^Household ventilation were assessed by frequency of opening windows, where the frequency under 3 times per week was categorized as " Rarely or never” and the frequency 3 times per week or over was categorized as “Everyday or occasionally”

### Association between residential proximity to major roadways and hearing impairment

The results of logistic regression analysis are given in Table 2. In the model adjusted for age and sex, compared with participants with residential proximity to major roadways > 300 m, the odds ratios (ORs) (95% CI) were 1.02 (0.92, 1.19), 1.06 (0.97, 1.17), 1.18 (1.06, 1.33) and 1.14 (1.02, 1.27), in those residing 200 to 300 m, 101 to 200 m, 50 to 100 m, and 50 m, respectively. The significance level of the associations became constant after stepwise adjustment of a set of covariates. In the fully-adjusted Model 4, the effect of residential proximity to major roadways on HI became more conspicuous for those dwelling < 50 m (OR 1.12, 95% CI: 1.01–1.31) and 50 to 100 m (OR 1.15, 95% CI: 1.07–1.34) than those living > 300 m away from the major roadways.

**Table 2:** Association of residential distance to major roadways with hearing status

Model	Horizontal distance to the major roadways (m)					
	< 50	50–100	101–200	201–300	> 300	P for trend
Unadjusted model	1.13 (1.03, 1.25)	1.17 (1.05, 1.32)	1.06 (0.96, 1.18)	1.04 (0.92, 1.17)	Ref.	0.005
Model 1	1.14 (1.02, 1.27)	1.18 (1.06, 1.33)	1.06 (0.97, 1.17)	1.02 (0.92, 1.19)	Ref.	0.002
Model 2	1.12 (1.02, 1.24)	1.14 (1.02, 1.30)	1.04 (0.94, 1.16)	1.02 (0.90, 1.17)	Ref.	0.023
Model 3	1.13 (1.02, 1.28)	1.14 (1.01, 1.29)	1.05 (0.85, 1.20)	1.03 (0.88, 1.21)	Ref.	0.014
Model 4	1.12 (1.01, 1.31)	1.15 (1.07, 1.34)	1.07 (0.96, 1.24)	1.02 (0.89, 1.28)	Ref.	< 0.001

^a^ Model 1: Adjusting for age, and sex

^b^ Model 2: Further adjusting for residency, education level, marital status, smoking status, drinking status, physical activity, leisure activity, dietary diversity, cognitive function, and BMI based on Model 1

^c^ Model 3: Further adjusting for household ventilation, continuous CO concentration, continuous PM_2.5_, continuous NO_2_, monthly average temperature based on Model 2

^d^ Model 4: Further adjusting for five kinds of diseases (diabetes, hypertension, heart disease, stroke and cancer

### Subgroup analysis

**Fig. 2:**
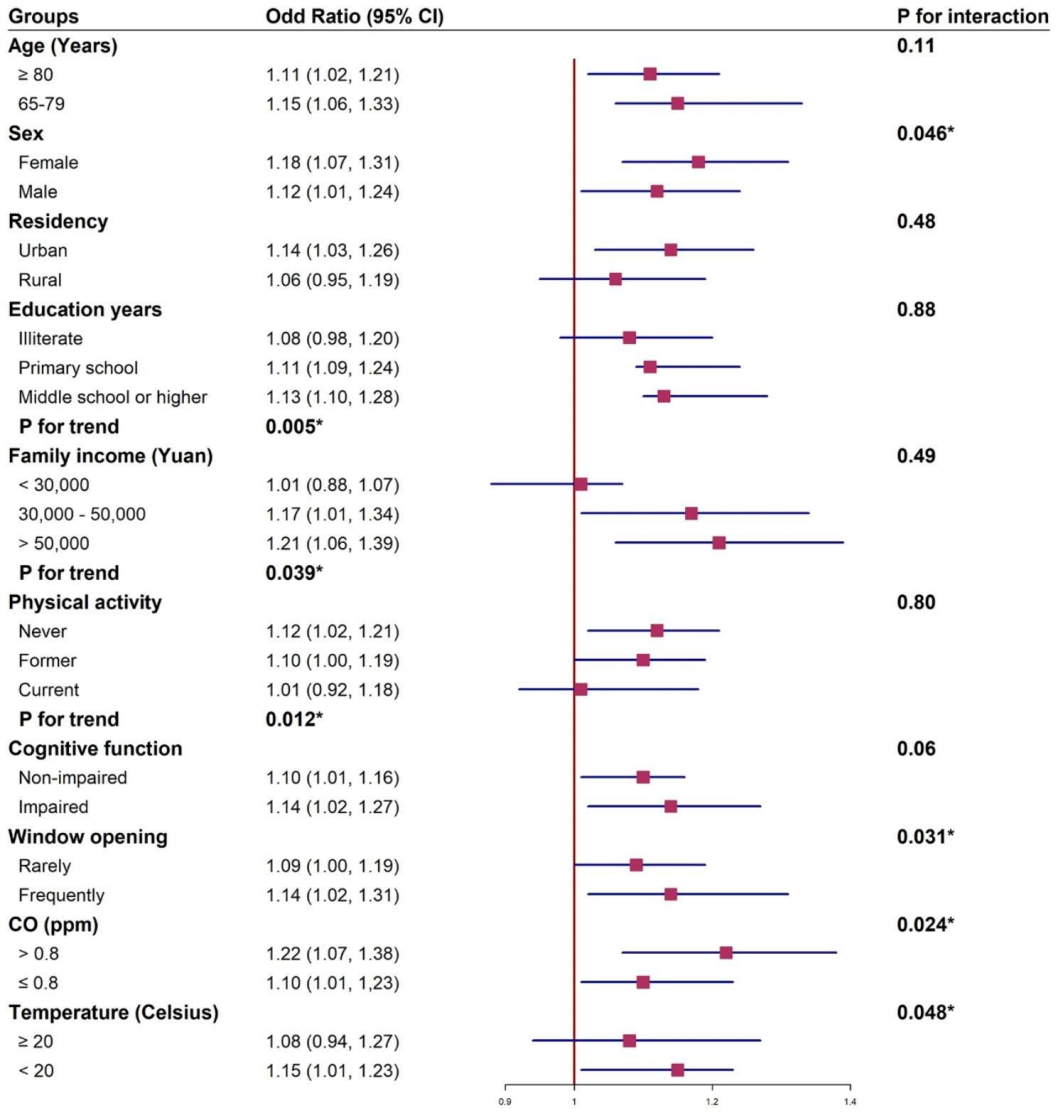
Association of residential distance to the major roadway (≤ 100 m vs. >100 m) with hearing status among subgroups. The association was assessed using Model 4 (Adjusting for age, sex, residency, education years, marital status, smoking status, drinking status, physical activity, leisure activity, dietary diversity, cognitive function, BMI, household ventilation, continuous CO concentration, continuous PM_2.5_, continuous NO_2_, monthly average temperature, and five kinds of diseases [diabetes, hypertension, heart disease, stroke and cancer])

Figure 2 shows the ORs of the association between residential proximity to major roadways and HI in ten pre-defined subgroups (in terms of age, sex, residency, education years, annual family income, cognitive function, physical activity, frequency of window-opening, annual average CO, and monthly temperature), analyzed as separate models for each subgroup with full adjustment as in Model 4. The negative effect of residential proximity to major roadways on hearing status were observed across all subgroups, while in some groups, the effect was not significant. The significant interaction persisted between binary residential distance to major roadways and sex (women vs. men, 1.18 [1.07, 1.31] vs. 1.12 [1.01, 1.24]), frequency of window-opening (Rarely vs. Frequently, 1.09 [1.00, 1.19] vs. 1.14 [1.02, 1.31]), annual CO concentration (> 0.8 ppm vs. ≤ 0.8 ppm, 1.22 [1.07, 1.38] vs. 1.10 [1.01, 1,23]), and monthly temperature (≥ 20℃ vs. < 20℃, 1.08 [0.94, 1.27] vs. 1.15 [1.01, 1.23]).

### Three-way interaction analysis

**Fig. 3:**
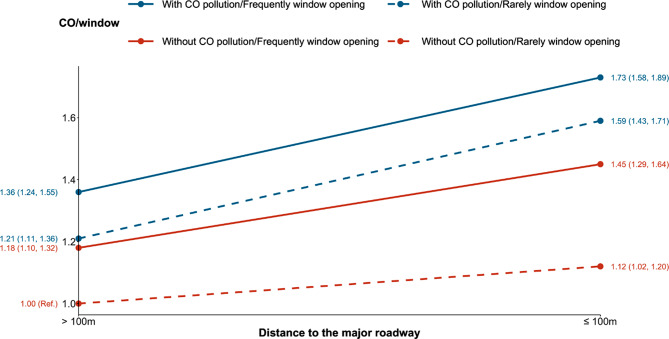
Three-way interaction of distance to nearest major roadway, CO pollution, and household ventilation on hearing impairment

The *t*-test revealed no correlation between window-opening and CO concentration, confirming the independence of each factors in the three-way interaction analysis (*P* = 0.081). In order to determine whether the effect of residential proximity to major roadways on HI differed in terms of frequency of window opening and CO concentration, we first established two models to assess the interaction between residential proximity to major roadways and windows opening or CO concentration, respectively. The results showed that interactions existed between residential proximity to major roadways and windows opening or CO concentration respectively (Figure A1). To further understand this interaction, we performed a three-way interaction analysis to evaluate the impact of CO concentration, window-opening and residential proximity on HI. The results showed that there were synergistic modifying effects of residential proximity, window-opening, CO concentration on HI. The groups exposed to CO pollution and opening windows frequently had the highest OR (1.73 [1.58, 1.89]), while the groups without exposure to CO pollution and opening windows rarely had the lowest OR (1.12 [95% CI: 1.02–1.20]) (Fig. 3).

### Sensitivity analysis

The magnitude and significance level of the effect of residential proximity to major roadways on HI remain unchanged after excluding participants who relocated within five years (n = 711, Table A3), those with severe cognitive impairment (*n* = 2,961, Table A4), and those with diabetes (*n* = 1,493), hypertension (*n* = 6,013), heart disease (*n* = 2,969), stroke (*n* = 1,753) and cancer (*n* = 452) (Table A5). We performed a robust Poisson regression to test the robustness of Model 4, and compared participants living < 300 m with their counterparts living > 300 m away from major roadways. The risk ratios were 1.01 (95% CI: 0.97, 1.03) for the distance of 200 to 300 m, 1.01 (95% CI: 0.98, 1.04) for the distance between 101 and 200 m, 1.04 (95% CI: 1.01, 1.06) for the distance between 50 and 100 m, and 1.03 (95% CI: 1.01, 1.05) for the distance < 50 m. The interpretation of the GLM results still applied although the magnitude of the effects became smaller compared to the logistic regression analysis (Table A6). The inclusion of age as a binomial term in all analyses yielded results consistent with the primary analysis (Table A7).

## Discussion

This population-based cohort study included 13,775 Chinese adults aged 65 years or over, and our results revealed an exposure-response relationship between residential proximity to major roadways and HI. The odds of HI were significantly increased in individuals living within 100 m away from major roadways compared to those living farther. Furthermore, the associations seemed stronger among participants exposed to CO pollution (> 0.8 ppm) and those opening windows frequently. To the best of our knowledge, this is the first epidemiological study conducted in a large sample, to evaluate the association between residential proximity to major roadways and HI in older adults.

Although no prior studies examined the relationship between residential proximity to roadways and HI in older adults, some researchers looked into the association between certain components of residential proximity to roadways, such as the link between traffic-related noise and HI [7, 35, 36]. A key pathological change of noise-related trauma is degeneration of cochlea, including the loss of outer hair cells in the organ of Corti, progressive Wallerian degeneration [37], vasoconstriction and reduced blood flow in inner ear [17]. A prior laboratory-based study of human participants had linked traffic noise to a higher risk of hearing loss [38]. Another study involving 46 policemen working on busy highways revealed a hypothetical relationship between road noise and HI [39]. Because of the scarcity of land due to the rapid urbanization in China and other low and middle incomes countries (LMICs), more and more major roadways and highways are being built close to residential areas, thereby exposing the residents to traffic-related noises. A WHO report noted that older people are more likely to perceive the negative impact of low-level road noise [45 dB(A)] [40]. In addition, because they are more restricted in mobility and outdoor activity, older adults tend to be more confined to their residential areas [41]. If they live closer to major roadways, they expose more to road noise than other age groups who spend less time there. Our study was conducted in a cohort of Chinese older adults geographically scattered over a large area, making it possible to extrapolate our results to populations in LMICs similar to China. The findings can inform the policy-making about noise-abatement in the city development and healthy ageing in other LMICs.

Apart from traffic noise, exposure to traffic-related air pollution could also be a possible mechanism underlying this association. Traffic-related air pollutants are a mixture of particulate matters (PM2.5 and PM10); CO, etc., which mechanistically promotes oxidative stresses involved in the development of HI [42]. Prior studies have shown that vehicle exhaust from the combustion of hydrocarbons is the most important source of ambient CO, which could cause the elevation of auditory thresholds and auditory sensitivity [42–44]. The aforementioned findings provided some clues to possible mechanisms of how residential proximity to major roadways results in HI. Our study found that the association between residential distance to roadways and HI was more pronounced in individuals exposed to CO (> 0.8 ppm) pollution and opening windows frequently. Our findings were consistent with the results of previous studies. One previous study conducted in Taiwan reported that the risk of sensorineural hearing loss was 45% (95% CI: 31 − 59%) higher in participants exposed to high-level CO concentration (> 0.76 ppm), than in those exposed to low CO levels (< 0.61 ppm), the findings being generally coincident with our results. In addition, we also observed a conspicuous adverse effect in the subjects who opened windows frequently (> 3 times per week) [45]. One plausible explanation was that the more frequent window opening allows more traffic air pollutants to enter the living area. Thus, living near major roadways with frequent windows opening might, in turn, exert a negative effect on HI.

Although the interaction was not statistically significant, our results suggested a significantly stronger association between living near major roadways and HI in participants enjoying a higher socioeconomic status (e.g., higher levels of education and incomes) [46]. Prior studies demonstrated that individuals living in proximity of major roads were generally in brackets of lower socioeconomic status (SES) in Western countries, which is opposite to the situation [16, 47], i.e., people who live closer to major roads tended to have received more education and to be in higher family income brackets in our study population. Another study conducted in China reported the same result [26]. A plausible explanation is that residential proximity to major roadways might be a potential indicator of better educational resources and convenient transportation due to under urbanization in China and other LMICs. Our results were less likely to be confounded by SES because a range of covariates related to SES were adjusted for SES in the study. However, we strongly suggested that the effect of SES being different from Western countries should be taken into account when evaluating the relationship between residential distance to major roadways and hearing function in China and other LMICs.

A major concern we had when planning this study was the acceptability of self-reported hearing status in our analysis. Generally, the assessment of individuals’ hearing status relied/relies on pure-tone audiometry [48]. Audiometry is the gold standard for evaluation of hearing loss, but large-scale use of the procedure is operationally infeasible due to such restraints as large number of trained staff, soundproof booths and equipment [49]. Thus, self-reporting may be a workable alternative. Furthermore, considering expenses and time involved, self-reporting can be a quick and cheap way to assess hearing function, especially in epidemiological studies [50]. A previous study suggested that the use of pure tone audiometry yielded similar results in term of the sensitivity to those of self-reporting [51]. Previously, a series of studies using data from CLHLS have explored the association between HI with multiple variables, confirming the robustness of self-reported HI in CLHLS [24, 25]. Additionally, we adjusted for a range of covariates that might interfere with the reliability of self-reported HI and performed several steps of sensitivity analyses to further enhance the reliability of our findings.

Our study was based on a representative sample and data were fully adjusted for potential confounders, rendering it possible to generalize our results to other LMICs. Nonetheless, the study is subject to several limitations. Frist, the study was of cross-sectional design, and, hence, we could not infer a causal relationship between residential proximity to major roadways and HI. Second, hearing status was measured by self-reporting, which might result in self-reporting bias. Third, we used self-reported distance to the major roadway as an approximation of residential distance to the major roadway. Hence recall biases were inevitable. Furthermore, the subjective interpretation of major roads might vary among individuals. Nevertheless, we have reduced this subjectivity to some extent by furnishing detailed instructions regarding what qualifies as a major roadway. Lastly, since we did not adjust for occupational information in all analyses, we don’t know, with any certainty, the impact of occupational noise exposure on hearing function.

## Conclusion

The present study was among the first to confirm an inverse exposure-response relationship between residential proximity to major roadways and HI, and provided evidence that exposure to CO pollution and opening windows frequently might synergistically strengthen this association. Because of fast urbanization and migration to the cities, more and more people are exposed to residence-related pollutants. Given residence is a modifiable factor, staying away from major roadways may be beneficial at personal, family and community levels. Our findings might promote city planners to consider keeping housing development away from the most heavily trafficked major roadways and inform policy-making about welfare of the older population in terms of hearing protection.

### Electronic supplementary material

Below is the link to the electronic supplementary material.

Supplementary Material 1: Detailed descriptions and definitions of covariates in current study. STROBE Statement. Characteristics of the study participants by the different groups of horizontal distance to the major roadways (n = 13,775). Sensitivity Analysis by excluding participants who changed residential addresses within five years (n = 711). Sensitivity Analysis by excluding participants whose MMSE score were lower than 18 (n = 2,961). Sensitivity Analysis by excluding participants with five kinds diseases (diabetes, hypertension, heart disease, stroke and cancer). Sensitivity Analysis by applying robust Poisson regression to determine association of residential distance to the major roadways with hearing impairment. Association of residential distance to major roadways with hearing status, adjusting for age as a binomial term. Correlations of independent variables and covariates with hearing impairment. Interaction of distance to nearest major roadway with CO pollution and household ventilation on hearing impairment, respectively. Supplemental References.

## Data Availability

The datasets used in the present study are openly accessed in the CLHLS from Peking University Open Research Data Platform (10.18170/DVN/WBO7LK).

## Abbreviations

HI: hearing impairment
CLHLS: the Chinese Longitudinal Healthy Longevity Survey
TRNP: traffic-related noise pollution
TRAP: traffic-related air pollution
CI: confidential Interval
OR: odds ratio
CO: carbon monoxide
SES: socioeconomic status
LMICs: low and middle-income countries
