# Supplementary material for: Residential proximity to major roadways and hearing impairment in Chinese older adults: a population-based study
Source: BMC Public Health. 2023 Dec 8;23:2462. doi: 10.1186/s12889-023-17433-6 (PMC10709848; doi:10.1186/s12889-023-17433-6)
Supplement: Supplementary file 1 — Supplementary Material 1: Detailed descriptions and definitions of covariates in current study. STROBE Statement. Characteristics of the study participants by the different groups of horizontal distance to the major roadways (n = 13,775). Sensitivity Analysis by excluding participants who changed residential addresses within five years (n = 711). Sensitivity Analysis by excluding participants whose MMSE score were lower than 18 (n = 2,961). Sensitivity Analysis by excluding participants with five kinds diseases (diabetes, hypertension, heart disease, stroke and cancer). Sensitivity Analysis by applying robust Poisson regression to determine association of residential distance to the major roadways with hearing impairment. Association of residential distance to major roadways with hearing status, adjusting for age as a binomial term. Correlations of independent variables and covariates with hearing impairment. Interaction of distance to nearest major roadway with CO pollution and household ventilation on hearing impairment, respectively. Supplemental References. [file 12889_2023_17433_MOESM1_ESM.docx]

*Appendix*

**Appendix Methods:** Detailed descriptions and definitions of covariates in current study

**Table A1.** STROBE Statement—checklist of items that should be included in reports of observational studies

**Table A2.** Characteristics of the study participants by the different groups of horizontal distance to the major roadways (n = 13,775).

**Table A3.** Sensitivity Analysis by excluding participants who changed residential addresses within five years (n = 711): Odds Ratio and 95% Confidential Interval of hearing impairment associated with residential distance to nearest major Roadway.

**Table A4.** Sensitivity Analysis by excluding participants whose MMSE score were lower than 18 (n = 2,961): Odds Ratio and 95% Confidential Interval of hearing impairment associated with residential distance to nearest major Roadway.

**Table A5.** Sensitivity Analysis by excluding participants with five kinds diseases (diabetes, hypertension, heart disease, stroke and cancer): Odds Ratio and 95% Confidential Interval of hearing impairment associated with residential distance to nearest major Roadway.

**Table A6.** Sensitivity Analysis by applying robust Poisson regression to determine association of residential distance to the major roadways with hearing impairment.

**Table A7.** Association of residential distance to major roadways with hearing status, adjusting for age as a binomial term.

**Table A8.** Correlations of independent variables and covariates with hearing impairment.

**Figure A1**. Interaction of distance to nearest major roadway with CO pollution and household ventilation on hearing impairment, respectively

**Supplemental References**

**Appendix Methods:**

**Detailed descriptions and definitions of covariates including in analysis in the present study**

Age was calculated according to self-reported birth date, based on Chinese lunar calendar dates, and converted to Georgian calendar dates. Marital status was divided as “currently married and living with spouse” or others (widowed, separated, divorced, or never married). Education level was grouped according to years of schooling 0, 1-6, and >6 years as “Illiterate”, “Primary school”, and “Middle school or higher”, respectively. Current residence was dichotomized as “urban areas” or “rural areas”. Smoking status was grouped as “Never”, “Former”, and “Current”, a similar approach was applied to define the drinking status and physical activity. Dietary diversity was calculated by intake frequency of eight food (vegetables, fruits, legumes and their products, nuts, meat, eggs, fish, dairy and its products) and categorized as “Low”, “Moderate”, and “High”. Family annual income was classified as “<30,000 Yuan”, “30,000 - 50,000 Yuan”, and “≥50,000 Yuan”. Frequency of opening windows was assessed by asking annual times of windows opening per week during past year, and was dichotomized as “Rarely” and “Frequently”. Body mass index (BMI) was calculated as weight in kilograms divided by height in meters squared. Leisure activity score was calculated by eight kinds of activities (whether a respondent did gardening, practiced Tai Chi, participated in square dance, raised poultry or pets, reading, playing Mahjong or cards, listening to the radio or watching TV, and participating in community social activities) and we scored each activity 1 for ‘never’, 2 for ‘sometimes’ 3 for ‘almost every day’; The score ranged from 8 to 24 with higher score indicate more leisure activities and low social and leisure activity level was defined by the score less than 14. Cognitive function was assessed using the Chinese version of The Mini-Mental State Examination (MMSE) during each survey through a home-based interview. The Chinese MMSE is consist of 24 components encompassing seven subdomains: orientation; naming foods; registration of three words; attention and calculation; copy a figure; recall; and language. The MMSE scores ranged from 0 to 30, with a lower score indicating worse cognitive function. The cognitive function was dichotomized as “Impaired” and “Non-impaired” with a cut-off score 25. Five types of common chronic condition were considered as covariates in the additional adjustment: diabetes, hypertension, heart disease, stroke and cancer.

Historical PM_2.5_ concentration and CO concentration were estimated from satellite remote-sensing measurements and outputs from a chemical transport model (CTM) of air pollution emission inventories. Satellite sensors, such as the Moderate Resolution Imaging Spectroradiometer, retrieve the column concentrations of aerosols from the earth's surface to the top of the atmosphere by measuring electromagnetic signals. Satellite-based annual estimates of air pollutants have been applied in many health-related studies at the national and global scales [1, 2]. The subjects in the CLHLS study could only be geo-coded to each participant's county code due to confidential reason. Therefore, we first pooled the data into city-level averages by matching the pixels of a regular grid with a map of China's prefectures, and further calculated the monthly averages. We utilized the PM_2.5_ and CO concentration averages during the 12 months preceding the surveyed months as the exposure values. We also obtained gridded estimates of temperature with an original resolution of 0.1° × 0.1° by fusing the satellite measurements of land surface temperatures, in-situ observations, and simulations from a weather-forecast research model [3]. The three types of temperature values were assembled by day using a universal kriging approach. City-level monthly averages for temperature data were also calculated for each record before the regression analyses.

**Additional Notes to the statistical plan**

Multiple imputation (MI) was used to impute our raw dataset. The MI is based on chained equations, which are commonly used for longitudinal studies. As it uses information about baseline demographics as well as previous time points to predict missing values, multiple imputation assumes that data are missing at random, that is, that missingness is related to observed data.

**Supplemental References**

[1] Y.X. Zheng, T. Xue, Q. Zhang, et al. Air quality improvements and health benefits from China's clean air action since 2013. Environ Res Lett, 12 (11) (2017)

[2] N. Goyal, M. Karra, D Canning. Early-life exposure to ambient fine particulate air pollution and infant mortality: pooled evidence from 43 low-and middle-income countries Int J Epidemiol (2019).

[3] T. Xue, T. Zhu, Y.X. Zheng, Q Zhang. Declines in mental health associated with air pollution and temperature variability in China. Nat Commun, 10 (2019)

**Table A1**. STROBE Statement—checklist of items that should be included in reports of observational studies

|  | Item No. | Recommendation | Page  No. | Relevant text from manuscript |
| --- | --- | --- | --- | --- |
| **Title and abstract** | 1 | (*a*) Indicate the study’s design with a commonly used term in the title or the abstract | 1 | A population-based study |
|  |  | (*b*) Provide in the abstract an informative and balanced summary of what was done and what was found | 2-3 | residential proximity to major roadways was significantly associated with an increased exposure-response risk of HI in Chinese older adults. Exposure to CO pollution and opening windows frequently might intensify the relations. |
| Introduction | | | |  |
| Background/rationale | 2 | Explain the scientific background and rationale for the investigation being reported | 6 | it is a public priority to identify and take measures to avoid its potential risk factors to lower the burden of HI. To date, however, no studies have specifically evaluated whether living proximity to major roadways is associated with HI. |
| Objectives | 3 | State specific objectives, including any prespecified hypotheses | 7 | (i) whether residential proximity to major roadways (a proxy of exposure to traffic) was associated with HI in an exposure-response manner among Chinese older adults; (ii) whether the association was modified by other factors. |
| Methods | | | |  |
| Study design | 4 | Present key elements of study design early in the paper | 8 | We used data from the CLHLS, an ongoing program which has been implemented since 1998 with follow-ups every 2 - 3 years. |
| Setting | 5 | Describe the setting, locations, and relevant dates, including periods of recruitment, exposure, follow-up, and data collection | 8-10 | In the present study, we used data of the 2018 wave of CLHLS, involving 15,874 participants. |
| Participants | 6 | (*a*) *Cohort study*—Give the eligibility criteria, and the sources and methods of selection of participants. Describe methods of follow-up  *Case-control study*—Give the eligibility criteria, and the sources and methods of case ascertainment and control selection. Give the rationale for the choice of cases and controls  *Cross-sectional study*—Give the eligibility criteria, and the sources and methods of selection of participants | 8 | First, we excluded 95 participants aged under 65 years. Then, participants with congenital deafness (n = 15) and sudden deafness (n = 150) were removed from our analysis. Additionally, considering the interference of ototoxic drugs on the hearing status, participants who were taking ototoxic medications were excluded from the main analysis (n = 11). Next, data on hearing status and residential proximity to major roadways were collected. After exclusion of 178 participants without data on hearing status, 1519 without residential information, and 131 without key covariables (cognitive function, as assessed by Chinese version of the Mini-Mental State Examination [MMSE]), 13,775 participants aged 65 years or over remained. The flow chart of study population is shown in Figure 1. |
|  |  | (*b*) *Cohort study*—For matched studies, give matching criteria and number of exposed and unexposed  *Case-control study*—For matched studies, give matching criteria and the number of controls per case | / |  |
| Variables | 7 | Clearly define all outcomes, exposures, predictors, potential confounders, and effect modifiers. Give diagnostic criteria, if applicable | 8-10 | 2.2 Residential proximity to major roadways;  2.3 Assessment of hearing impairment (HI) |
| Data sources/ measurement | 8* | For each variable of interest, give sources of data and details of methods of assessment (measurement). Describe comparability of assessment methods if there is more than one group | 8-10 |  |
| Bias | 9 | Describe any efforts to address potential sources of bias | 9 |  |
| Study size | 10 | Explain how the study size was arrived at | 8 |  |

Continued on next page

| Quantitative variables | 11 | Explain how quantitative variables were handled in the analyses. If applicable, describe which groupings were chosen and why | / |  |
| --- | --- | --- | --- | --- |
| Statistical methods | 12 | (*a*) Describe all statistical methods, including those used to control for confounding | 10-11 | We established four multivariable logistic regression models to evaluate the association between residential proximity to major roadways and HI |
|  |  | (*b*) Describe any methods used to examine subgroups and interactions | 11 |  |
|  |  | (*c*) Explain how missing data were addressed | 10 | Multiple imputation (MI) was used to impute missing data |
|  |  | (*d*) *Cohort study*—If applicable, explain how loss to follow-up was addressed  *Case-control study*—If applicable, explain how matching of cases and controls was addressed  *Cross-sectional study*—If applicable, describe analytical methods taking account of sampling strategy | / |  |
|  |  | (*e*) Describe any sensitivity analyses | 11 | We conducted several sensitivity analyses to verify the robustness of the results of Model 4. |
| Results | | | | |
| Participants | 13* | (a) Report numbers of individuals at each stage of study—eg numbers potentially eligible, examined for eligibility, confirmed eligible, included in the study, completing follow-up, and analysed | / |  |
|  |  | (b) Give reasons for non-participation at each stage | / |  |
|  |  | (c) Consider use of a flow diagram | 12 |  |
| Descriptive data | 14* | (a) Give characteristics of study participants (eg demographic, clinical, social) and information on exposures and potential confounders | 12 | The median age of participants was 85.0 years (range of 65 - 117 years) and more than 50% of them (55.9%) were women. The prevalence of self-reported HI stood at 38.3%. A total of 2494 participants (18.1%) lived within 50 m from a major roadway; 2128 (15.5%) resided 50 to 100 m, 1438 (10.4%) 101 to 200 m, 1305 (9.5%) 201 to 300 m, and 6410 (46.5%) > 300 m away from major roadways. |
|  |  | (b) Indicate number of participants with missing data for each variable of interest | 13 |  |
|  |  | (c) *Cohort study*—Summarise follow-up time (eg, average and total amount) | / |  |
| Outcome data | 15* | *Cohort study*—Report numbers of outcome events or summary measures over time | */* |  |
|  |  | *Case-control study—*Report numbers in each exposure category, or summary measures of exposure | */* |  |
|  |  | *Cross-sectional study—*Report numbers of outcome events or summary measures | 13 |  |
| Main results | 16 | (*a*) Give unadjusted estimates and, if applicable, confounder-adjusted estimates and their precision (eg, 95% confidence interval). Make clear which confounders were adjusted for and why they were included | 12 | In the model adjusted with age and sex, compared with participants with residential proximity to major roadways > 300 m, the odds ratios (ORs) (95% CI) were 1.02 (0.92, 1.19), 1.06 (0.97, 1.17), 1.18 (1.06, 1.33) and 1.14 (1.02, 1.27), for those residing 200 m to 300 m, 101 m to 200 m, 50 m to 100 m, and 50 m, respectively. |
|  |  | (*b*) Report category boundaries when continuous variables were categorized | 13 |  |
|  |  | (*c*) If relevant, consider translating estimates of relative risk into absolute risk for a meaningful time period | / |  |

Continued on next page

| Other analyses | 17 | Report other analyses done—eg analyses of subgroups and interactions, and sensitivity analyses | 14 | Figure 2 shows the ORs by residential proximity to major roadways for ten pre-defined subgroups |
| --- | --- | --- | --- | --- |
| Discussion | | | | |
| Key results | 18 | Summarise key results with reference to study objectives | 14-15 | This population-based cohort study included 13,775 Chinese adults aged 65 years or over, and our results revealed an exposure-response relationship between residential proximity to major roadways and HI. |
| Limitations | 19 | Discuss limitations of the study, taking into account sources of potential bias or imprecision. Discuss both direction and magnitude of any potential bias | 18 | Our study was based on a representative sample and data were fully adjusted for potential confounders, rendering it possible to generalize our results to other LMICs. Nonetheless, the study is subject to several limitations. |
| Interpretation | 20 | Give a cautious overall interpretation of results considering objectives, limitations, multiplicity of analyses, results from similar studies, and other relevant evidence | 18 | Our findings supported city planners to consider locating housing developments away from the most heavily trafficked major roadways on a public policy level which can yield benefits to healthy aging. |
| Generalisability | 21 | Discuss the generalisability (external validity) of the study results | 18-19 |  |
| Other information | |  | | |
| Funding | 22 | Give the source of funding and the role of the funders for the present study and, if applicable, for the original study on which the present article is based | 20 |  |

*Give information separately for cases and controls in case-control studies and, if applicable, for exposed and unexposed groups in cohort and cross-sectional studies.

**Note:** An Explanation and Elaboration article discusses each checklist item and gives methodological background and published examples of transparent reporting. The STROBE checklist is best used in conjunction with this article (freely available on the Web sites of PLoS Medicine at http://www.plosmedicine.org/, Annals of Internal Medicine at http://www.annals.org/, and Epidemiology at http://www.epidem.com/). Information on the STROBE Initiative is available at www.strobe-statement.org.

**Table A2.** Characteristics of the study participants by the different groups of horizontal distance to the major roadways (n = 13,775).

|  | **Distance From Residence to Major Roadways (m)** | | | | | | *P*_trend_ |
| --- | --- | --- | --- | --- | --- | --- | --- |
|  | **<50**  **(n = 2494)** | **50-100**  **(n = 2128)** | **101-200**  **(n = 1438)** | **201-300**  **(n = 1305)** | **>300**  **(n = 6410)** | **Total**  **(n = 13,775)** | Value |
| **Marital status**, N (%) |  |  |  |  |  |  | 0.076 |
| Currently married and living with spouse | 1025 (41.1) | 848 (39.8) | 589 (41.0) | 504 (38.6) | 2507 (39.1) | 5473 (39.7) |  |
| Widowed, divorced, or never married | 1469 (58.9) | 1280 (60.2) | 849 (59.0) | 801 (61.4) | 3903 (60.9) | 8302 (60.3) |  |
| **Smoking status**, N (%) |  |  |  |  |  |  |  |
| Never | 1725 (69.2) | 1527 (71.8) | 1027 (71.4) | 944 (72.3) | 4556 (71.1) | 9779 (71.0) | 0.031 |
| Former | 409 (16.4) | 311 (14.6) | 229 (15.9) | 200 (15.3) | 962 (15.0) | 2111 (15.3) | 0.26 |
| Current | 360 (14.4) | 290 (13.6) | 182 (12.7) | 161 (12.4) | 892 (13.9) | 1885 (13.7) | 0.015 |
| **Drinking status**, N (%) |  |  |  |  |  |  |  |
| Never | 1852 (74.3) | 1616 (75.9) | 1105 (76.8) | 967 (74.1) | 4673 (72.9) | 10,213 (74.1) | 0.17 |
| Former | 312 (12.5) | 226 (10.6) | 141 (9.8) | 146 (11.2) | 802 (12.5) | 1627 (11.8) | 0.33 |
| Current | 330 (13.2) | 286 (13.5) | 192 (13.4) | 192 (14.7) | 935 (14.6) | 1935 (14.1) | 0.28 |
| **Dietary diversity ^b^**, N (%) |  |  |  |  |  |  |  |
| Low | 910 (36.5) | 695 (32.7) | 455 (31.6) | 438 (33.6) | 2483 (38.7) | 4981 (36.2) | 0.401 |
| Moderate | 1418 (56.8) | 1233 (57.9) | 847 (58.9) | 765 (58.6) | 3608 (56.3) | 7871 (57.1) | 0.277 |
| High | 166 (6.7) | 200 (9.4) | 136 (9.5) | 102 (7.8) | 319 (5.0) | 923 (6.7) | 0.195 |
| **BMI categories, kg/m^2^**, N (%) |  |  |  |  |  |  |  |
| **< 18.5** | 490 (19.6) | 393 (18.5) | 271 (18.8) | 254 (19.5) | 1229 (19.2) | 2637 (19.1) | 0.266 |
| **18.5 to < 24** | 1232 (49.4) | 1083 (50.9) | 669 (46.5) | 654 (50.1) | 3243 (50.6) | 6881 (50.0) | 0.104 |
| **≥ 24** | 772 (31.0) | 652 (30.6) | 498 (34.7) | 397 (30.4) | 1938 (30.2) | 4257 (30.9) | 0.204 |
| **Diabetes**, N (%) | 281 (11.3) | 312 (14.7) | 219 (15.2) | 150 (11.5) | 531 (8.3) | 1493 (10.8) | <0.001 |
| **Hypertension**, N (%) | 1080 (43.3) | 1019 (47.9) | 728 (50.6) | 605 (46.3) | 2581 (40.3) | 6013 (43.7) | <0.001 |
| **Heart disease**, N (%) | 577 (23.1) | 560 (26.3) | 397 (27.6) | 324 (24.8) | 1111 (17.3) | 2969 (21.6) | <0.001 |
| **Stroke**, N (%) | 334 (13.4) | 295 (13.9) | 245 (17.0) | 167 (12.8) | 712 (11.1) | 1753 (12.7) | <0.001 |
| **Cancer**, N (%) | 88 (3.5) | 89 (4.2) | 67 (4.7) | 44 (3.4) | 164 (2.6) | 452 (3.3) | 0.001 |

**Table A3.** Sensitivity Analysis by excluding participants who changed residential addresses within five years (*n* = 711): Odds Ratio and 95% Confidential Interval of hearing impairment associated with residential distance to nearest major Roadway.

| **Model** | **Horizontal distance to the major roadways (m)** | | | | |
| --- | --- | --- | --- | --- | --- |
|  | **<50** | **50-100** | **101-200** | **201-300** | **>300** |
| Model 1 | 1.12 (1.01, 1.24) | 1.15 (1.02, 1.30) | 1.05 (0.95, 1.17) | 1.04 (0.92, 1.18) | Ref. |
| Model 2 | 1.11 (1.00, 1.23) | 1.13 (1.01, 1.29) | 1.04 (0.91, 1.14) | 1.02 (0.90, 1.18) | Ref. |
| Model 3 | 1.12 (1.02, 1.25) | 1.14 (1.01, 1.29) | 1.04 (0.92, 1.18) | 1.02 (0.88, 1.19) | Ref. |
| Model 4 | 1.12 (1.01, 1.31) | 1.15 (1.07, 1.34) | 1.07 (0.96, 1.24) | 1.03 (0.89, 1.28) | Ref. |

Model 1: Adjusting for age, and sex;

Model 2: Further adjusting for residency, education level, marital status, smoking status, drinking status, physical activity, leisure activity, dietary diversity, cognitive function, and BMI based on Model 1;

Model 3: Further adjusting for household ventilation, continuous CO concentration, continuous PM_2.5_, continuous NO_2_, monthly average temperature based on Model 2;

Model 4: Further adjusting for five kinds of diseases (diabetes, hypertension, heart disease, stroke and cancer) based on Model 3.

**Table A4**. Sensitivity Analysis by excluding participants whose MMSE score were lower than 18 (*n* = 2,961): Odds Ratio and 95% Confidential Interval of hearing impairment associated with residential distance to nearest major Roadway.

| **Model** | **Horizontal distance to the major roadways (m)** | | | | |
| --- | --- | --- | --- | --- | --- |
|  | **<50** | **50-100** | **101-200** | **201-300** | **>300** |
| Model 1 | 1.14 (1.04, 1.33) | 1.15 (1.02, 1.30) | 1.04 (0.94, 1.17) | 1.04 (0.92, 1.19) | Ref. |
| Model 2 | 1.12 (1.01, 1.22) | 1.14 (1.01, 1.29) | 1.03 (0.90, 1.15) | 1.03 (0.91, 1.20) | Ref. |
| Model 3 | 1.13 (1.03, 1.26) | 1.16 (1.03, 1.27) | 1.05 (0.91, 1.19) | 1.04 (0.87, 1.19) | Ref. |
| Model 4 | 1.12 (1.02, 1.30) | 1.15 (1.05, 1.33) | 1.03 (0.89, 1.26) | 1.02 (0.85, 1.30) | Ref. |

Model 1: Adjusting for age, and sex;

Model 2: Further adjusting for residency, education level, marital status, smoking status, drinking status, physical activity, leisure activity, dietary diversity, cognitive function, and BMI based on Model 1;

Model 3: Further adjusting for household ventilation, continuous CO concentration, continuous PM_2.5_, continuous NO_2_, monthly average temperature based on Model 2;

Model 4: Further adjusting for five kinds of diseases (diabetes, hypertension, heart disease, stroke and cancer) based on Model 3.

**Table A5**. Sensitivity Analysis by excluding participants with five kinds diseases (diabetes, hypertension, heart disease, stroke and cancer): Odds Ratio and 95% Confidential Interval of hearing impairment associated with residential distance to nearest major Roadway.

| **Diseases** | **Number of excluding participants** | **Horizontal distance to the major roadways (m)** | | | | |
| --- | --- | --- | --- | --- | --- | --- |
|  |  | **<50** | **50-100** | **101-200** | **201-300** | **>300** |
| Diabetes | 1,493 | 1.04 (1.01, 1.06) | 1.05 (1.01, 1.08) | 1.01 (0.98, 1.03) | 0.99 (0.97, 1.04) | Ref. |
| Hypertension | 6,013 | 1.08 (1.00, 1.12) | 1.10 (1.02, 1.15) | 1.03 (0.96, 1.06) | 1.02 (0.90, 1.04) | Ref. |
| Heart diseases | 2,969 | 1.12 (1.01, 1.16) | 1.14 (1.02, 1.20) | 1.05 (0.97, 1.11) | 1.01 (0.94, 1.06) | Ref. |
| Stroke | 1,753 | 1.09 (0.98, 1.18) | 1.12 (1.01, 1.20) | 1.04 (0.98, 1.09) | 1.03 (0.97, 1.07) | Ref. |
| Cancer | 452 | 1.12 (1.02, 1.31) | 1.15 (1.07, 1.34) | 1.06 (0.96, 1.25) | 1.02 (0.90, 1.28) | Ref. |

The associations were assessed using Model 4 (Adjusting for age, sex, residency, education years, marital status, smoking status, drinking status, physical activity, leisure activity, dietary diversity, cognitive function, BMI, household ventilation, continuous CO concentration, continuous PM_2.5_, continuous NO2, monthly average temperature, and five kinds of diseases [diabetes, hypertension, heart disease, stroke and cancer]).

**Table A6.** Sensitivity Analysis by applying robust Poisson regression to determine association of residential distance to the major roadways with hearing impairment.

| **Model** | **Horizontal distance to the major roadways (m)** | | | | |
| --- | --- | --- | --- | --- | --- |
|  | **<50** | **50-100** | **101-200** | **201-300** | **>300** |
| Model 1 | 1.02 (1.01, 1.06) | 1.05 (1.02, 1.10) | 1.02 (0.97, 1.05) | 1.02 (0.98, 1.06) | Ref. |
| Model 2 | 1.05 (1.00, 1.09) | 1.07 (1.01, 1.13) | 1.01 (0.95, 1.06) | 1.00 (0.94, 1.08) | Ref. |
| Model 3 | 1.04 (1.02, 1.08) | 1.05 (1.01, 1.09) | 1.00 (0.96, 1.05) | 0.98 (0.97, 1.03) | Ref. |
| Model 4 | 1.02 (1.01, 1.05) | 1.03 (1.02, 1.05) | 1.01 (0.97, 1.03) | 1.02 (0.98, 1.04) | Ref. |

Model 1: Adjusting for age, and sex;

Model 2: Further adjusting for residency, education level, marital status, smoking status, drinking status, physical activity, leisure activity, dietary diversity, cognitive function, and BMI based on Model 1;

Model 3: Further adjusting for household ventilation, continuous CO concentration, continuous PM_2.5_, continuous NO_2_, monthly average temperature based on Model 2;

Model 4: Further adjusting for five kinds of diseases (diabetes, hypertension, heart disease, stroke and cancer) based on Model 3.

**Table A7**. Association of residential distance to major roadways with hearing status, adjusting for age as a binomial term.

| **Model** | **Horizontal distance to the major roadways (m)** | | | | | |
| --- | --- | --- | --- | --- | --- | --- |
|  | **<50** | **50-100** | **101-200** | **201-300** | **>300** | **P for trend** |
| Model 1 | 1.12 (1.02, 1.29) | 1.17 (1.04, 1.37) | 1.05 (0.95, 1.21) | 1.00 (0.93, 1.18) | Ref. | 0.009 |
| Model 2 | 1.13 (1.03, 1.30) | 1.16 (1.05, 1.37) | 1.03 (0.96, 1.20) | 1.03 (0.92, 1.18) | Ref. | 0.028 |
| Model 3 | 1.13 (1.02, 1.30) | 1.15 (1.03, 1.34) | 1.04 (0.88, 1.18) | 1.03 (0.89, 1.17) | Ref. | 0.039 |
| Model 4 | 1.17 (1.04, 1.31) | 1.20 (1.02, 1.33) | 1.11 (0.94, 1.19) | 1.09 (0.95, 1.19) | Ref. | 0.011 |

^a^ Model 1: Adjusting for age, and sex;

^b^ Model 2: Further adjusting for residency, education level, marital status, smoking status, drinking status, physical activity, leisure activity, dietary diversity, cognitive function, and BMI based on Model 1;

^c^ Model 3: Further adjusting for household ventilation, continuous CO concentration, continuous PM_2.5_, continuous NO_2_, monthly average temperature based on Model 2;

^d^ Model 4: Further adjusting for five kinds of diseases (diabetes, hypertension, heart disease, stroke and cancer

**Table A8**. Correlations of independent variables and covariates with hearing impairment.

| **Groups** | **Model 1**  **Sex-age** | **Model 2** | **Model 3** | **Model 4** |
| --- | --- | --- | --- | --- |
| **Residential distance to the major roadway** |  |  |  |  |
| >300 m | Ref. | Ref. | Ref. | Ref. |
| 201-300 m | 1.02 (0.92, 1.19) | 1.02 (0.90, 1.17) | 1.03 (0.88, 1.21) | 1.02 (0.89, 1.28) |
| 101-200 m | 1.06 (0.97, 1.17) | 1.04 (0.94, 1.16) | 1.05 (0.85, 1.20) | 1.07 (0.96, 1.24) |
| 50-100 m | 1.18 (1.06, 1.33) | 1.14 (1.02, 1.30) | 1.14 (1.01, 1.29) | 1.15 (1.07, 1.34) |
| < 50 m | 1.14 (1.02, 1.27) | 1.12 (1.02, 1.24) | 1.13 (1.02, 1.28) | 1.12 (1.01, 1.31) |
| **Age** | 1.15 (1.07, 1.22) | 1.18 (1.04, 1.27) | 1.10 (1.02, 1.16) | 1.09 (1.00, 1.17) |
| **Sex** |  |  |  |  |
| Women | Ref. | Ref. | Ref. | Ref. |
| Men | 0.92 (0.83, 0.99) | 0.88 (0.79, 0.93) | 0. 96 (0.91, 1.03) | 0.98 (0.90, 1.04) |
| **Residence** |  |  |  |  |
| Rural |  | Ref. | Ref. | Ref. |
| Urban |  | 1.09 (1.02, 1.18) | 1.05 (0.98, 1.11) | 1.08 (1.03, 1.16) |
| **Education level** |  |  |  |  |
| Illiterate |  | Ref. | Ref. | Ref. |
| Primary school |  | 0.91 (0.88, 0.97) | 0.93 (0.89, 0.99) | 0.95 (0.90, 1.02) |
| Middle school or higher |  | 0.96 (0.85, 0.98) | 0.91 (0.87, 0.95) | 0.93 (0.89, 0.96) |
| **Marital status** |  |  |  |  |
| Currently married and living with spouse |  | Ref. | Ref. | Ref. |
| Widowed, divorced, or never married |  | 1.11 (1.07, 1.14) | 1.12 (1.08, 1.17) | 1.14 (1.10, 1.19) |
| **Smoking status** |  |  |  |  |
| Never |  | Ref. | Ref. | Ref. |
| Former |  | 0.94 (0.91, 1.07) | 0.91 (0.88, 1.04) | 0.91 (0.88, 1.05) |
| Current |  | 0.88 (0.84, 1.01) | 0.85 (0.83, 1.01) | 0.86 (0.84, 1.00) |
| **Drinking status** |  |  |  |  |
| Never |  | Ref. | Ref. | Ref. |
| Former |  | 0.96 (0.93, 1.02) | 0.95 (0.92, 1.01) | 0.94 (0.91, 1.00) |
| Current |  | 0.94 (0.93, 1.03) | 0.92 (0.90, 1.02) | 0.94 (0.90, 1.01) |
| **Physical activity** |  |  |  |  |
| Never |  | Ref. | Ref. | Ref. |
| Former |  | 0.96 (0.89, 0.98) | 0.94 (0.90, 0.98) | 0.95 (0.89, 0.97) |
| Current |  | 0.90 (0.88, 0.95) | 0.91 (0.88, 0.96) | 0.90 () |
| **Leisure activity** |  |  |  |  |
| Low |  | Ref. | Ref. | Ref. |
| High |  | 0.92 (0.87, 0.98) | 0.95 (0.81, 1.00) | 0.94 (0.88, 1.04) |
| **Dietary diversity** |  |  |  |  |
| Low |  | Ref. | Ref. | Ref. |
| Moderate |  | 0.94 (0.91, 0.97) | 0.94 (0.91, 1.02) | 0.98 (0.89, 1.07) |
| High |  | 0.85 (0.77, 0.90) | 0.86 (0.74, 0.89) | 0.94 (0.91, 0.97) |
| **Cognitive function** |  |  |  |  |
| Without |  | Ref. | Ref. | Ref. |
| With |  | 1.26 (1.15, 1.31) | 1.22 (1.13, 1.29) | 1.18 (1.10, 1.27) |
| **BMI** |  |  |  |  |
| **18.5 – 24** |  | Ref. | Ref. | Ref. |
| **< 18.5** |  | 0.92 (0.87, 0.98) | 0.91 (0.86, 0.98) | 0.96 (0.83, 1.01) |
| **≥ 24** |  | 1.20 (1.10, 1.31) | 1.23 (1.13, 1.34) | 1.18 (1.08, 1.27) |
| **Window opening** |  |  |  |  |
| Rarely or never |  |  | Ref. | Ref. |
| Everyday or occasionally |  |  | 1.07 (1.02, 1.13) | 1.08 (1.03, 1.13) |
| **CO** |  |  | 1.14 (1.08, 1.20) | 1.16 (1.07, 1.21) |
| **PM_2.5_** |  |  | 1.08 (0.97, 1.12) | 1.05 (0.96, 1.11) |
| **NO_2_** |  |  | 1.04 (0.92, 1.10) | 1.02 (0.87, 1.07) |
| **Temperature** |  |  | 0.93 (0.90, 1.02) | 0.95 (0.92, 1.03) |
| **Diabetes** |  |  |  |  |
| Without |  |  |  | Ref. |
| With |  |  |  | 1.20 (1.14, 1.27) |
| **Hypertension** |  |  |  |  |
| Without |  |  |  | Ref. |
| with |  |  |  | 0.94 (0.90, 1.03) |
| **Heart disease** |  |  |  |  |
| Without |  |  |  | Ref. |
| With |  |  |  | 1.02 (0.97, 1.08) |
| **Stroke** |  |  |  |  |
| Without |  |  |  | Ref. |
| With |  |  |  | 1.08 (1.01, 1.11) |
| **Cancer** |  |  |  |  |
| Without |  |  |  | Ref. |
| With |  |  |  | 1.10 (1.05, 1.15) |

Model 1: Adjusting for age, and sex;

Model 2: Further adjusting for residency, education level, marital status, smoking status, drinking status, physical activity, leisure activity, dietary diversity, cognitive function, and BMI based on Model 1;

Model 3: Further adjusting for household ventilation, continuous CO concentration, continuous PM_2.5_, continuous NO_2_, monthly average temperature based on Model 2;

Model 4: Further adjusting for five kinds of diseases (diabetes, hypertension, heart disease, stroke and cancer) based on Model 3.


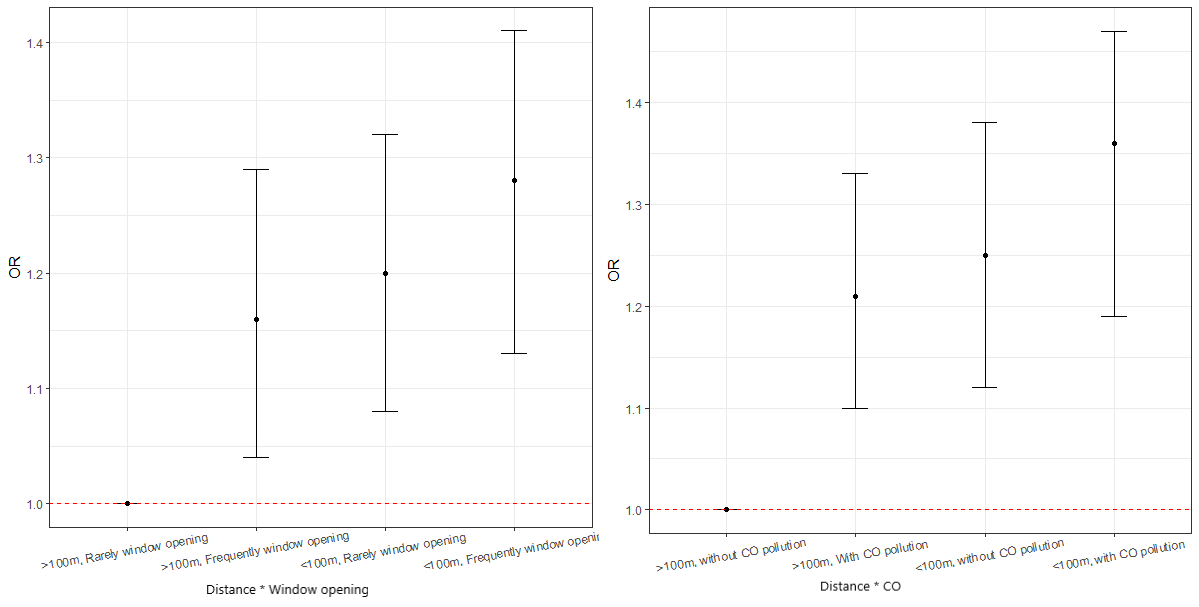


**Fig.A1**. Interaction of distance to nearest major roadway with CO pollution and household ventilation on hearing impairment, respectively.

CO pollution was defined as continuous CO concentration higher than 0.8 ppm. The frequency of opening window was categorized as two groups: Frequently window opening (more than 3 times per week) *vs*. Rarely window opening (3 times or less per week).

The associations were assessed using Model 4 (Adjusting for age, sex, residency, education years, marital status, smoking status, drinking status, physical activity, leisure activity, dietary diversity, cognitive function, BMI, household ventilation, continuous CO concentration, continuous PM_2.5_, continuous NO_2_, monthly average temperature, and five kinds of diseases [diabetes, hypertension, heart disease, stroke and cancer]).

OR: Odds Ratio
